# Differential Radiomodulating Action of *Olea europaea* L. cv. Caiazzana Leaf Extract on Human Normal and Cancer Cells: A Joint Chemical and Radiobiological Approach

**DOI:** 10.3390/antiox11081603

**Published:** 2022-08-19

**Authors:** Severina Pacifico, Pavel Bláha, Shadab Faramarzi, Francesca Fede, Katarina Michaličková, Simona Piccolella, Valerio Ricciardi, Lorenzo Manti

**Affiliations:** 1 Dipartimento di Scienze e Tecnologie Ambientali Biologiche e Farmaceutiche, Università della Campania “Luigi Vanvitelli”, 81100 Caserta, Italy; 2 Istituto Nazionale di Fisica Nucleare-Sezione di Napoli, 80126 Napoli, Italy; 3 Department of Plant Production and Genetics, Faculty of Agriculture, Razi University, Kermanshah 67149-67346, Iran; 4 Dipartimento di Fisica “E. Pancini”, Università degli Studi di Napoli Federico II, 80126 Napoli, Italy

**Keywords:** *Olea europaea* L. cv. Caiazzana, oleacein, ionising radiation, cancer, radiotherapy, normal tissue, radioprotection, radiosensitization, radiomodulation

## Abstract

The identification of a natural compound with selectively differential radiomodulating activity would arguably represent a valuable asset in the striving quest for widening the therapeutic window in cancer radiotherapy (RT). To this end, we fully characterized the chemical profile of olive tree leaf polyphenols from the Caiazzana cultivar (OLC), autochthonous to the Campania region (Italy), by ultra-high-performance liquid chromatography–high-resolution mass spectrometry (UHPLC-HR-MS). Oleacein was the most abundant molecule in the OLC. Two normal and two cancer cells lines were X-ray-irradiated following 24-h treatment with the same concentration of the obtained crude extract and were assessed for their radioresponse in terms of micronucleus (MN) induction and, for one of the normal cell lines, of premature senescence (PS). Irradiation of pre-treated normal cells in the presence of the OLC reduced the frequency of radiation-induced MN and the onset of PS. Conversely, the genotoxic action of ionising radiation was exacerbated in cancer cells under the same experimental conditions. To our knowledge, this is the first report on the dual action of a polyphenol-rich olive leaf extract on radiation-induced damage. If further confirmed, these findings may be pre-clinically relevant and point to a substance that may potentially counteract cancer radioresistance while reducing RT-associated normal tissue toxicity.

## 1. Introduction

External beam-based radiotherapy (RT) is one of the most effective strategies used to treat cancer by ionising radiation (IR). However, RT-associated acute and late-occurring normal tissue reactions may significantly affect a patient’s life quality [1,2] and increase the risk of RT-induced secondary cancers [3,4], limiting de facto the curative dose that can be safely administered to the tumour [5]. Moreover, acquired [6,7] or intrinsic [8,9] cancer cell radioresistance correlates with failure in achieving tumour local control in RT, leading to poor prognosis, recurrence, and/or metastatization. Hence, although technological development and improvements in treatment planning systems have greatly increased accuracy in dose delivery to the tumour target and normal tissue sparing, late RT toxicity (e.g., fibrosis, cardiac events, cognitive impairment) remains a burden for adult cancer survivors and a long-term health hazard in paediatric patients [10]. In this context, the pursuit of radiomodulating agents is an actively sought strategy for widening the therapeutic index when administered in conjunction with IR as they can be a valuable aid if able to increase normal tissue protection and/or IR tumouricidal effectiveness. In fact, the IR research program of the National Cancer Institute classified, according to administration timing, agents with IR-protective properties in three categories: (a) protection, (b) mitigation, and (c) therapeutic agents [11,12]. Analogously, many compounds that selectively modify cancer cell radioresponse have long been studied, such as halogenated pyrimidines and hypoxic cell sensitizers [13], in order to shift the tumour-control curve to lower doses without affecting the normal-tissue complication curve. This would result in an increase in tumour-control probability for a given level of adverse effects. However, currently available radioprotectors/radiosensitizers have several limitations, including high toxicity per se and costs. Moreover, for a radiomodulating compound to offer a practical gain in RT, it has to show a differential effect between normal tissues and tumours, as it would be of no avail to use a drug that increases the radiosensitivity of tumours and normal tissues alike, nor would it be therapeutically sound to mitigate radiation-induced damage in the two compartments to the same extent. The general failure of synthetic compounds to act as selective radioprotectors and the difficulty in finding effective radiosensitizers with low normal-tissue toxicity has driven researchers to focus on natural substances with radiomodulating potential and several botanicals, which could be less expensive than synthetic ones, have been screened for their radioprotective or radiosensitizing activity [14,15,16]. Plant-derived polyphenols in particular have gained considerable attention in the long-standing quest for intrinsically low-toxic radioprotecting/radiosensitizing drugs [12,17]. Free radical scavenging, anti-inflammation properties, facilitation of repair processes, and the regeneration of hematopoietic cells are the main mechanisms attributable to natural radioprotectors. In particular, since most of the IR damage arises from the interaction of IR-induced free radicals with biomolecules, natural substances, such as curcumin, chlorogenic acids, and different flavonoids could serve as radioprotectors, being able to combine with or prevent the formation of free radicals [18]. On the other hand, the anti-cancer activity of natural radiosensitizers has been correlated with their ability to inhibit the intracellular glutathione (GSH) redox buffering metabolism [19], to abrogate radiation-induced cell-cycle checkpoints that favour damage repair [20], to downregulate expression of genes implicated in cancer cell proliferation and resistance to radiation-induced apoptosis such as COX-2 [21], and to counteract tumour progression and migration by inhibiting pro-inflammation nuclear factor kappa B (NF-kB) transcription factor and disrupting a number of signalling pathways implicated in uncontrolled proliferation and enhanced angiogenesis, as reviewed in [16].

Moreover, the attractive double-edged potential of pure polyphenols or polyphenol-enriched extracts to act as both radiosensitizing and radioprotective agents would arguably hold pre-clinical significance and have a significant impact on the general prognosis of tumours refractory to radiation treatment [17,20,22]. *Olea europaea* L. leaf is a rich source of phenols and polyphenols, whose radioprotective potential was marginally investigated in pre-IR and post-IR treatments [23]. Anticlastogenic and antiradical activities of an olive leaf extract, constituted by 24.5% in oleuropein, 1.5% in hydroxytyrosol, and by almost 3% in flavone-7-glucosides and 1% in verbascoside, were found. On the other hand, a radiosensitizing action by pure oleuropein on cancer cell radioresponse was determined in nasopharyngeal carcinoma [24], highlighting the need of acquiring new insights in olive leaf radio-nutraceutical properties. Hence, taking into account the abundance and uniqueness of olive trees varieties in the Campania Region [25], we investigated the chemistry and radiobiology of the leaves of *O. europaea* cultivar “Caiazzana”, autochthonous of the Campania region, whose name derives from Caiazzo, near Caserta [26]. To this purpose, an alcoholic olive leaf extract was prepared and chemically characterized by means of high-resolution tandem mass spectrometry; its antiradical capability was assessed through DPPH and ABTS tests. Furthermore, normal and cancer cell lines were exposed in vitro to graded doses of X-rays following treatment with, and in the presence of, a given concentration of the extract, whose effect on their radioresponse was evaluated in terms of the modulation of radiation-induced cyto-genotoxicity. Specifically, all cell lines were assayed for the induction of DNA damage-associated micronuclei (MN) and one normal cell line for the onset of radiation-induced premature senescence (PS). At the investigated concentration, the extract consistently reduced MN frequency in normal cells but increased their occurrence in cancer cells after exposure to X-rays. Treatment with, and irradiation in the presence of, the extract also abated PS. To the best of our knowledge, this is the first time that the phytochemical profile and the radiomodulating activity of an olive leaf extract have been investigated, unveiling a specifically selective action that expressed itself, at the same extract concentration, as a concomitant mitigation or exacerbation of radiation-induced damage in normal and cancer cells, respectively.

## 2. Materials and Methods

### 2.1. Olive Leaf Collection and Extraction

*O. europaea* L. cv. Caiazzana leaves were collected at the experimental site of CREA (Consiglio per la ricerca in agricoltura e l’analisi dell’economia agraria) in Casagiove (Caserta, Italy; 41°04′25.0″ N 14°18′59.4″ E, alt. 68 a.s.l) on 2 September 2020. Immediately after harvesting, they were transferred to the Food Chemistry Laboratory of University of Campania “Luigi Vanvitelli” and lyophilized by the FTS-System Flex-dryTM instrument (SP Scientific, Stone Ridge, NY, USA) for 48 h.

An aliquot of the plant matrix was pulverized by using a mortar and a pestle and then extracted through Ultrasound Assisted Maceration (UAM), using pure ethanol as extractant with a solid:liquid ratio equal to 1:10 (*w*:*v*). At the end each sonication cycle (3 in total; 30 min each), the sample was filtered and then dried by a rotary evaporator ((Heidolph Hei-VAP Advantage, Schwabach, Germany), obtaining a crude extract (OLC) with a yield of 27%.

### 2.2. Chemical Characterization: UV-Vis, HPLC-UV-DAD and UHPLC-ESI-TOF/MS

OLC UV-Vis spectrum was recorded by double beam, dual chopper Cary 100 spectrophotometer (Agilent, Milano, Italia) in the range of 200–800 nm.

HPLC-UV-DAD analyses were carried out on a 1260 Infinity II LC System (Agilent, Santa Clara, CA, USA) equipped with an Agilent G711A quaternary pump and a WR G7115A diode array detector. The separation was achieved on Phenyl-Hexyl Column (150 × 2.0 mm i.d., 3.0 μm particle size, Phenomenex, Torrance, CA, USA), with a gradient of water (A) and acetonitrile (B), both with 0.1% formic acid. Starting with 95% A, a linear gradient was followed to 85% A in 10 min, which then decreased to 75.0% A at 25 min, to 60% A at 40 min, to 30% A at 50 min, and, finally, to 5% A at 60 min. The mobile phase composition was maintained at 5% A for another 2.0 min, then returned to the starting conditions and was allowed to re-equilibrate for 2 min. The flow rate was set at 0.3 mL min^−1^, and the injection volume was 5.0 μL. UV detection was set at five different wavelengths (220, 280, 320, 340, and 360 nm).

UHPLC-HR-MS techniques were applied for a detailed profiling of OLC chemical constituents. To this purpose, a NEXERA UHPLC system (Shimadzu, Tokyo, Japan) was used with a Luna^®^ Omega C-18 column (1.6 μm particle size, 50 × 2.1 mm, Phenomenex). The separation took advantage of a linear gradient of water (A) and acetonitrile (B), both with 0.1% formic acid, as follows: 0–5 min, 5→15% B; 5–12.5 min, 15→25% B; 12.5–20 min, 25→40% B; 20–25 min, 40→70% B; held at 75% B for other 2 min. The %B reached 95% for a column clean-up step and then returned to the starting conditions re-equilibrated for 1 min. The total analysis time was 27 min, the flow rate was 0.5 mL/min, and the injection volume was 2.0 μL. HR-MS and MS/MS spectra were recorded in negative electrospray ionization (ESI) mode, using the AB SCIEX Triple TOF^®^ 4600 (AB Sciex, Concord, ON, Canada). The APCI probe was used for automated mass calibration in all scan functions using the Calibrant Delivery System (CDS). Non-targeted approach was developed, combining TOF-MS and MS/MS with Information Dependent Acquisition (IDA), consisting of a full scan TOF survey (accumulation time 250 ms, 100–1000 Da) and eight IDA MS/MS scans (accumulation time 100 ms, 80–800 Da). Other source and analyser parameters were the following: curtain gas (CUR) 35 psi, nebulizer gas (GS 1) 60 psi, heated gas (GS 2) 60 psi, ion spray voltage (ISVF) 4.5 kV, interface heater temperature (TEM) 600 °C, declustering potential (DP) −70 V, collision Energy (CE) −35 V, collision energy spread (CES) 10 V. The instrument was controlled by Analyst^®^ TF 1.7 software, while data processing was carried out using PeakView^®^ software version 2.2.

### 2.3. Radical Scavenging Capacity: DPPH and ABTS Tests

OLC was tested at 50, 25, 12.5, 6.25, and 3.125 μg/mL (final concentrations) vs. ABTS [2,2′-azinobis-(3-ethylbenzothiazolin-6-sulfonic acid)] radical cation and 2,2-diphenyl-1-picrylhydrazyl (DPPH) radical [27]. Trolox (4, 8, 16, 32 µM) was used as standard, and all recorded activities were compared to a blank sample, arranged in parallel. ABTS^●+^ was produced by mixing (2,2′-azinobis-(3-ethylbenzothiazolin-6-sulfonic acid); 7 mM) and potassium persulfate (K_2_S_2_O_8_; 2.45 mM) in the dark for 12 h. ABTS^●+^ was thus diluted with PBS (pH 7.4) to reach an absorbance of 0.7 at 734 nm and was allowed to react with the OLC concentrations. After 6 min, the absorbance was measured using a Wallac Victor3 spectrophotometer in reference to a blank. DPPH^●^ methanol solution (9.4 × 10^−5^ M), reacting with OLC at different concentrations, served to assess DPPH^●^ scavenging activity. The mixtures were stirred for 15 min, and the absorption was read at 517 nm by Wallac Victor3 spectrophotometer in reference to a blank. The results were expressed in terms of the percentage reduction in the initial radicals’ adsorption by tested samples. Trolox (4, 8, 16, 32 µM) was used as positive standard. All data were expressed as mean ± standard deviation (SD).

### 2.4. Fe (III) Reducing Power

The ability of OLC (at 50, 25, 12.5, 6.25, and 3.125 µg/mL final concentrations) to reduce the Fe^3+^ using a ferricyanide FRAP assay was evaluated according to PFRAP procedure [27]. The absorbance was measured at 700 nm. The increase in absorbance with reference to the blank was considered. Trolox (4, 8, 16, 32 µM) was used as positive standard. All data were expressed as mean ± standard deviation (SD).

### 2.5. Cell Cultures: Maintenance and Preparation

All cell lines employed in this work are commercially available and were either purchased or received as a gift. Human Umbilical Vein Endothelial Cells (HUVECs) were purchased from Lonza Ltd. (Basel, Switzerland) as cryopreserved ampules of pooled cells from donors at passage 1. After thawing, they were grown in EGM-2 Bullet Kit™ and routinely sub-cultured at a density between 2000 and 4000 cells/cm^2^ as per manufacturer’s instructions. All experiments were performed with cells between passage 3 and 5. The spontaneously immortalized, non-transformed human mammary epithelial MCF-10A cells and the primary prostate adenocarcinoma DU145 cells were kindly donated by Dr. P. Chaudhary (CCRCB, Queens University, Belfast, UK). As described in detail by Debnath et al., two DMEM/F12-based media were necessary for MCF-10A cells: one for optimal growth, enriched with 5% horse serum, Endothelial Growth Factor (20 ng/mL), hydrocortisone (0.5 mg/mL), insulin (10 mg/mL), and cholera toxin (100 ng/mL); the other, devoid of all supplements but rich in horse serum (20%), to be used only for the quenching of trypsin during routine sub-cultivation and cell counting dilutions. DU145 cells were grown in RPMI medium, complemented with 10% foetal bovine serum (FBS) and 1% of L-glutamine (L-Gln). Finally, human pancreatic epithelioid carcinoma PANC-1 cells (a gift from Dr. A. Facoetti, CNAO, Pavia, Italy) were cultured in a high-glucose (4.5 g/l) DMEM medium and supplemented with FBS and L-Gln as mentioned above. Penicillin/streptomycin was added (1%) to media for all cell lines except HUVECs. Cell lines were grown in standard tissue culture flasks maintained at 37 °C in a humidified atmosphere (95% air, 5% CO_2_).

To assess the radiomodulating properties of OLC, the water-soluble extract was added at a final concentration of 12.5 μL/mL to exponentially growing cells seeded at appropriate densities in T12.5 or T25 tissue culture flasks for senescence time-course experiments (around 1.5 × 10^4^ and 5 × 10^4^ cells/flask, respectively) or in Nunc™ (Thermo Fisher Scientific, Waltham, MA, USA) slide flasks (around 2 × 10^4^ cells/slide flask) for DNA damage evaluation by the CBMN assay. Cells were thus incubated for 24 h prior to exposure to radiation. Cells were irradiated in OLC-containing media. Immediately after irradiation, the medium was discarded, cells were rinsed thoroughly in Phosphate Buffer Saline (PBS) solution, and the samples were processed for evaluation of radiation-induced PS or MN formation, as detailed below. Non-OLC-treated cells were used as controls and subjected to the same experimental conditions.

### 2.6. Cell Irradiation

In all experiments, cells were exposed to 1-mm Cu-filtered X-rays generated by a radiogen tube (STABILIPAN, Siemens, Berlin, Germany) at 250 kVp at a dose rate of about 1.36 Gy/min at the Radiation Biophysics Laboratory, Physics Department, University of Naples Federico II. Dose uniformity was within 5% in a 15-cm-long square field as ensured by regular dosimetry performed with an Accu-Pro™ Radcal^®^ ionization chamber.

### 2.7. β-Galactosidase Assay for Quantification of Cellular Senescence in HUVECs

The occurrence of radiation-induced PS was assessed by the detection of senescence-associated β-galactosidase activity at pH 6.0 using a commercially available kit (Sigma-Aldrich, Merck KGaA, Darmstadt, Germany). After irradiation, HUVECs were placed back into the incubator (after eventually removing the extract-containing medium, as mentioned in Section 2.3): those seeded in T12.5 flasks were assayed after 24 or 48 h, while those irradiated in T25 flasks were left to grow for one week and then trypsinized and re-seeded in T12.5 tissue culture flasks. When attached after a few hours, they were processed as their early time counterparts. For all time points, glutaraldehyde-fixed cells were incubated in the β-gal-specific solution at 37 °C in the absence of CO_2_ overnight. Senescence-specific affinity for β-gal conferred a distinct greenish colour to cells when observed under bright field microscopy using a 10× magnification. The fraction of senescent cells was thus determined by the counting of random fields. Between 750 and 1000 cells were scored per time, dose, and treatment (OLC vs. non-OLC) conditions. The use of T12.5 flasks was the optimal compromise between having enough cells to analyze and economize the kit’s reagents.

### 2.8. Determination of Radiation-Induced DNA Damage

The genotoxic action of X-rays was evaluated by means of the Cytokines-Block MicroNucleus (CBMN) assay in all the cell lines. After irradiation, cells seeded onto slide flasks were treated for 24 h with 2.0 μg/mL of the actin-disrupting agent Cythocalasin B (CytB) that inhibits cytoplasmic furrow cleavage in dividing cells, thereby arresting them at the binucleated (BN) stage. In the CBMN assay, therefore, DNA damage manifests itself in the form of round DNA portions that failed to be incorporated in either of the daughter nuclei and is quantified by the occurrence of such micronuclei (MN) satisfying well-established morphological criteria in BN cells. After 24 h, cells were washed by PBS and then fixed by slowly adding a freshly prepared 4:1 Carnoy’s solution (Methanol; Acetic acid) that had been kept at −20 °C for at least 20 min. Fixation was also performed at −20 °C for 20 min before removing the fixative and breaking apart the slide flasks. These were air-dried for 24 h and then stained by 12–14 μL of 250 ng/mL DAPI/Antifade. Scoring was performed by an epi-fluorescence Zeiss Axioplan 2 imaging microscope with a 40× magnification objective. The frequency of MN/cell was determined according to the formula:

(1) $$\frac{{\mathrm{MN}}_{1}{+2\times \mathrm{MN}}_{2}{+3\times \mathrm{MN}}_{3}{+4\times \mathrm{MN}}_{4}}{\mathrm{BN}}$$

where MN_n_ is the number of BN cells with n MN and BN is the total number of BN cells scored. BN cells carrying more than 5 MN were extremely rare and mostly accompanied by aberrant cell morphology and were hence not included in the analysis. Between 500 and 1500 BN cells were scored according to the radiation dose for statistical robustness. Statistical significance was determined at the 95% confidence level using a two-sample *t*-test using SYSTAT (version 13.1, USA).

## 3. Results

A crude extract, hereinafter referred to as OLC, from *O. europaea* L. cv. Caiazzana leaves, was metabolically profiled for its polyphenolic content by an array of techniques aimed at compound separation and structural characterization. The putative ability by the thus profiled polyphenol-rich OLC, to afford in vitro normal tissue radioprotection and to increase tumour cell radiosensitivity, was subsequently tested by measuring its modulation of X-ray-induced cytogenetic damage in four cell lines, namely two of cancer and two of non-cancer origin.

### 3.1. Chemical Profiling of Olive Tree Leaf Polyphenols

**Figure 1:**
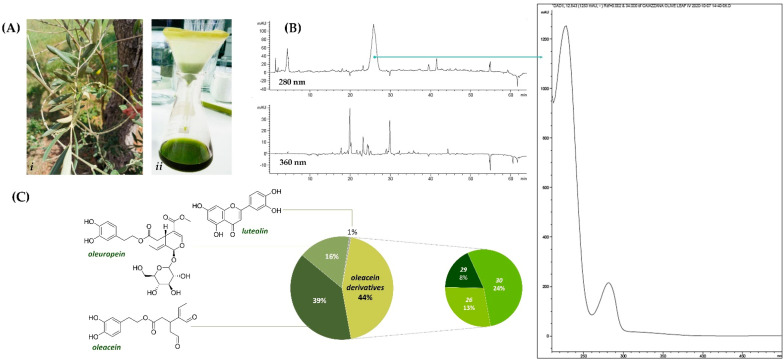
(**A**) Leaves of *O. europaea* cv. Caiazzana (i) and OLC extract therefrom (ii); (**B**) HPLC-UV chromatograms with the UV-DAD spectrum of a secoiridoid compound; (**C**) calculated amounts (%) of quantified compounds in OLC.

The experimental workflow applied in order to investigate the radiomodulating activity of olive leaf polyphenols for their radionutraceutical exploitation cannot disregard a rational and systematic chemical approach. Thus, with the aim to maximize the recovery of polyphenol compounds, leaves from *O. europaea* L. cv. Caiazzana (Figure 1A) were pulverized and underwent Ultrasound-Assisted Maceration (UAM) in ethanol. The alcoholic extract obtained was firstly evaluated by Ultraviolet-Visible (UV-Vis) spectroscopy, highlighting that, beyond typical phenol secoiridoids’ absorptions, bands relative to flavonoids, carotenoids (415 and 480 nm), and chlorophylls (670 nm) occurred. The High-Performance Liquid Chromatography–Ultraviolet (HPLC-UV) chromatograms, reported in Figure 1B, showed a main peak at 280 nm, whose Ultraviolet Diode Array Detection (UV-DAD) spectrum was in accordance with that of phenolic secoiridoids, with three main peaks detected at <210, 230, and 282 nm [28]. Furthermore, the HPLC-UV profile at 360 nm confirmed the presence of flavonoids, as this absorption could be due to B ring band I [29].

Based on this preliminary experimental evidence, a deep investigation of the chemical composition and relative content was carried out by means of Ultra High Performance Liquid Chromatography High Resolution Mass Spectrometry (UHPLC-HR-MS) techniques. In Table 1, all TOF-MS and TOF-MS/MS data were listed, while the Total Ion Current (TIC) and base peak chromatogram (BPC) are depicted in Figure S1, whereas quantitation data are depicted in Figure 1C.

**Figure 2:**
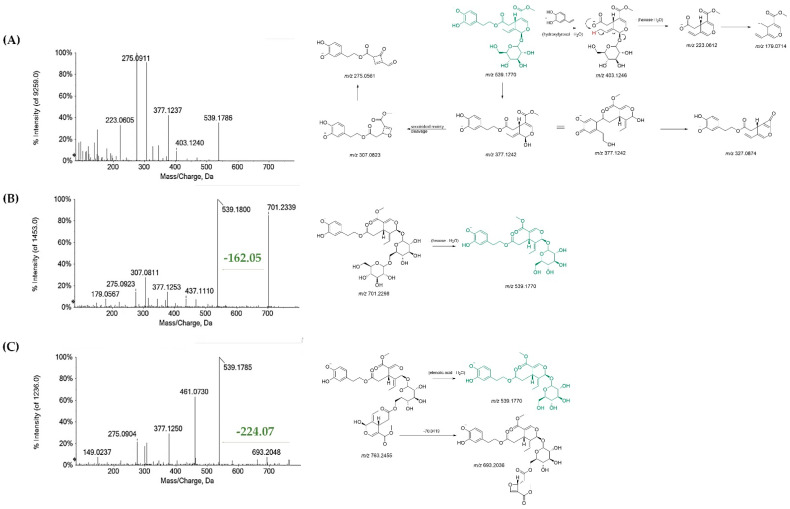
Tandem mass spectra and relative proposed comprehensive fragmentation pattern of (**A**) oleuropein (**22**); (**B**) oleuropein hexoside (**18**); (**C**) elenoyl oleuropein (**31**). Theoretical *m*/*z* values are reported under each structure.

Beyond an hexitol (**1**), likely mannitol, previously detected in water-stressed *O. europaea* leaves [30], and 12-hydroxyjasmonate sulfate (**5**), also commonly associated to plant stress and defense responses [31], quinic acid (**2**) [32], hydroxytyrosol (**3**), and its hexoside (**4**), were putatively identified. TOF-MS/MS spectra of both compounds **3** and **4** showed the fragment ion at *m*/*z* 123.046, likely formed by the loss of formaldehyde (Figure S2). Phenethyl primeveroside (**7**) and the ethyl-glucopyranosyloxy-oxopropylcyclohexaneacetic (**8**) were also identified [33]. Indeed, most of the OLC compounds consisted in phenolic secoiridoids. Oleuropein (**22**), which is one of the most representative constituents of olive tree organs and related products, by-products, and wastes [34], was detected at 10.183 min retention time. Although it was not the most abundant compound, it accounted for 16% of OLC metabolic composition (Figure 1C). The TOF-MS/MS experiment of oleuropein (**22**) allowed us to gain an insight into diagnostic fragment ions for its ready identification. In fact, the deprotonated molecular ion at *m*/*z* 539.1784 underwent neutral loss of the dehydrated hydroxytyrosol to provide the ion at *m*/*z* 403.1240 (elenolic acid glucoside). The latter, in turn, could lose the hexose unit to achieve the ion at *m*/*z* 223.0605 (elenolic acid). Furthermore, the deprotonated molecular ion underwent sugar loss, providing the ion at *m*/*z* 377.1237, which supplied the fragment at *m*/*z* 307.0839 following the secoiridoid moiety cleavage. Other important peaks in the TOF-MS/MS spectrum were rationalized, and their chemical structures are reported in Figure 2A.

Other phenolic secoiridoids were a monohexosyl oleuropein (**18**), with the [M-H]-ion at *m*/*z* 701.2287 (Figure 2B) [35] and lucidumoside B (**19**). The latter, previously isolated in other plants belonging to the same family, e.g., *Ligustrum lucidum* [36], was closely related to oleuropein. Another oleuropein derivative was compound **31**, whose deprotonated molecular ion was detected at *m*/*z* 763.2461 (Figure 2C). A thorough and systematic study of its high-resolution MS/MS spectrum, compared to those of the secoiridoids discussed herein, allowed us to tentatively identify it as an interesting oleuropein derivative, never described before, and characterized by the presence of a second elenolate moiety, likely linked to the sugar residue at its hydroxymethyl function (Figure S3).

**Figure 3:**
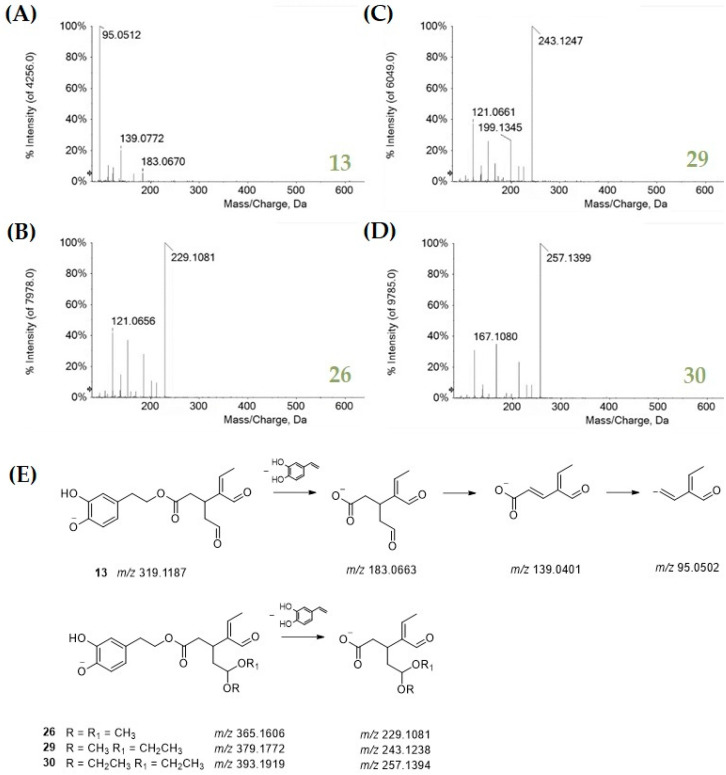
TOF-MS/MS spectra of (**A**) oleacein and its acetal derivatives **26** (**B**), **29** (**C**), and **30** (**D**). The proposed fragmentation pattern of all these compounds is in panel (**E**). Theoretical *m*/*z* values are reported under each structure.

The most abundant OLC compound was oleacein (**13**), accounting for 39%. This compound, also known as hydroxyoleocanthal, is a di-aldehydic derivative of oleuropein aglycone, with antioxidant, anti-inflammatory, anti-proliferative, and antimicrobial activities [37]. The TOF-MS/MS spectrum of the compound highlighted a diagnostic loss of 136 Da due to 4-vinylbenzene-1,2-diol from the hydroxytyrosol moiety (Figure 3A). This spectrometric feature was also found in the TOF-MS/MS spectra of compounds **26**, **29,** and **30** (Figure 3, panels B–E). The first one is likely an oleacein dimethyl acetal, whereas compounds **29** and **30** were the ethylmethyl and the diethyl acetal derivatives. These compounds, accounting for 44% of OLC (Figure 1), were reported as possible artifacts in virgin olive oils due to the oleacein interaction with polar solvents used for oil extraction and LC analysis methods [38,39].

Other minor compounds in OLC were verbascoside (**12**) [40] and the *p*-coumaroyl phenylethanoid glycoside isomers (**15** and **21**). The TOF-MS/MS spectra of all these compounds shared the loss of the hydroxycinnamoyl moiety to achieve the deprotonated glycosylated hydroxytyrosol and the dehydrated hydroxycinnamate as a less abundant fragment ion (Figure S3). Finally, several glycosylated flavonoids (**9**–**11**, **14**, **16**, **17**, **20**, **23**, **24**) were tentatively identified. Briefly, compounds **9**, **11**, **17,** and **23** shared luteolin as aglycone, and they differed in the number and/or the identity and/or glycosylation site of saccharide moieties (Figure S4). TOF-MS/MS spectra of the identified apigenin glycosides at *m*/*z* 577.1575(7) (**16**, **20**), diosmin (**24**) and rutin (**10**) showed the loss of a dehydrated rutinose (−308.11 Da). Luteolin, apigenin, and diosmetin also occurred in free form (**25**, **27**, **28**) (Figure S5). In particular, among flavonoids, luteolin was the most abundant, although its content was about only 1% in OLC (Figure 1). Quercetin deoxyhexoside (**14**) was also tentatively identified (Figure S4). The presence of almost all glycosides in this plant organ was thoroughly documented, among which flavone 7-*O*-glycosides are the most cited, together with rutin, as reported by Quirantes-Piné et al. [32] and references therein.

The less polar compound, eluting at 25.740 min retention time, was identified as the pentacyclic triterpenoid oleanolic acid (**32**). In fact, the [M-H]-ion, detected at *m*/*z* 455.3549, underwent decarboxylation and subsequent reduction to achieve the ion at *m*/*z* 407.3324, which in turn could be reduced to give the ion at *m*/*z* 405.3152 or undergo methane loss to provide the ion at *m*/*z* 373.2535 (Figure S6). The identification was confirmed by comparison with a pure commercial standard. Oleanolic acid was previously found to enhance the radiosensitizing effect on tumour cells. It was observed that MN frequencies in the hypoxic cells treated with oleanolic acid were augmented after irradiation compared with the cells without oleanolic acid treatment. The effect of the pentacyclic triterpenoid was ascribed to the reduction in intracellular GSH content and HIF-1α expression [41].

### 3.2. OLC Antiradical and Reducing Activities

**Figure 4:**
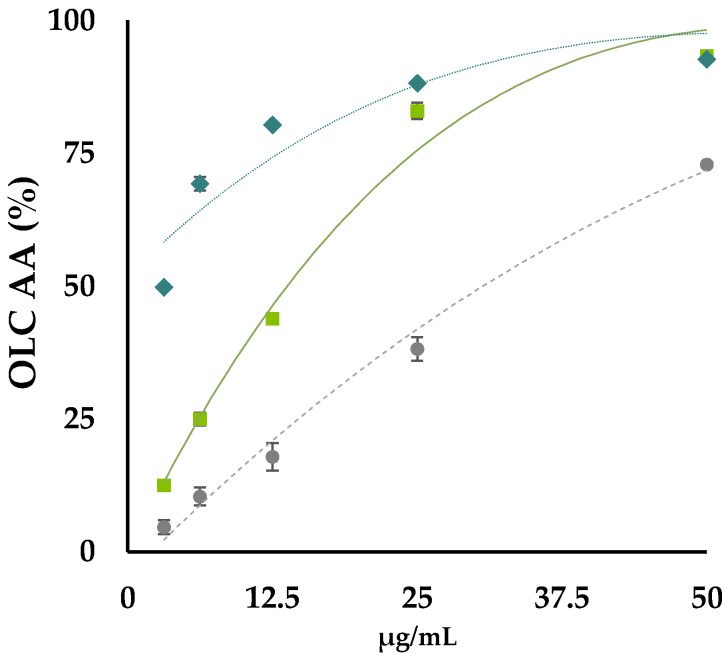
OLC antioxidant activity (AA%) vs. 2,2′-azino-bis(3-ethylbenzothiazoline)-6-sulfonic acid (ABTS) radical cation (♦), and vs. 2,2-diphenyl-1-picrylhydrazy (DPPH) radical (●). Fe (III) reducing power (RP) by PFRAP assay is also reported (■). Values reported are the mean ± SD of three independent measurements.

OLC underwent antiradical screening using as probes the radicals ABTS^●+^ [2,2′-azinobis-(3-ethylbenzothiazoline-6-sulfonic acid)] and DPPH^●^ (2,2-Diphenyl-1-picrylhydrazyl). The ID_50_ value, which is the dose of antioxidant species able to reduce by 50% the initial UV absorption of the radical probe, was calculated by plotting radical scavenging capability versus extract dose levels (Figure 4).

Data acquired allowed us to observe that OLC exerted a strongly dose-dependent radical scavenging capability. It was effective towards ABTS cation radical with an ID_50_ value equal to 13.5 μg/mL, whereas it was able to scavenge by 50% DPPH when it was at 26 μg/mL. The activity was comparable to that exerted by Trolox in the analogue water soluble of vitamin E, commonly used as a pure reference compound in antioxidant assays. Moreover, OLC exhibited a strong reducing power. In fact, it was also able to effectively reduce ferric ions when the lowest concentration was tested. The activity gradually increased, reaching the plateau at 25 μg/mL. Data acquired were in line with previous findings by Lins et al. [42], although no details about the chemical composition of the investigated extract were reported. The hydroxytyrosol moiety, which is shared by the largest part of the identified compounds, thanks to its electron donating capacity, reasonably allowed the scavenging effect, as well as the reducing power, to be exerted [25].

### 3.3. Selective OLC-Mediated Radiomodulating Effects in Normal and Cancer Cells

Preliminary tests by MTT (3-[4,5-dimethylthiazol-2-yl]-2,5 diphenyl tetrazolium bromide) assay were conducted on HUVECs and allowed us to identify the optimal OLC concentration of 12.5 μg/mL and pre-treatment time (24 h), as defined by the absence of significant metabolic alterations and cytotoxicity in unirradiated samples (data not shown). HUVECs were chosen because they are a particularly sensitive primary cell line. Such experimental conditions were then applied to all investigated cell lines. Their appropriateness was confirmed by the lack of any detectable difference in terms of the induction of cytogenetic damage per se between unirradiated OLC-treated cells and unirradiated non-OLC-treated cells, as shown below.

A clear and consistent differential action was exhibited by the OLC on the cellular radioresponse of cancer and normal cells. The 24-h treatment period followed by exposure to graded doses of the X-rays of cells in the presence of the extract at the concentration of 12.5 μg/mL resulted in a significant reduction in radiation-induced damage in the two normal cell lines (HUVEC and MCF-10A) while exacerbating its occurrence in the two cancer cell lines (DU145 and PANC-1) used in this study compared to non-OLC-treated irradiated samples.

#### 3.3.1. OLC Decreases Radiation-Induced Premature Senescence (PS) in HUVECs

**Figure 5:**
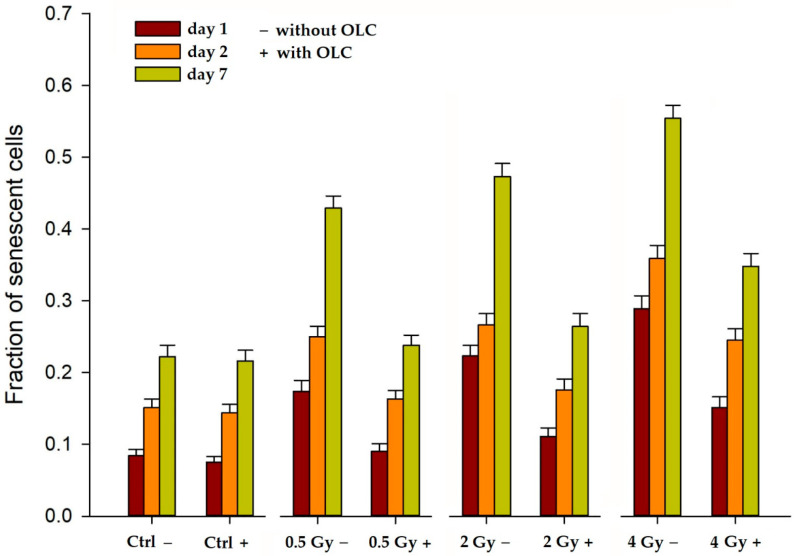
Time–course occurrence of X-ray-induced premature senescence (PS) in HUVECs in the presence (+ with OLC) and the absence of (− without OLC) of the extract. OLC mediates reduction of PS for all radiation doses at the three times assayed post-irradiation. Reported values refer to two separate experiments. Error bars refer to standard errors (SE) of the mean.

Pre-treatment with, and irradiation in the presence of, OLC resulted in a marked decrease in the onset of X-ray-induced PS in HUVECs, a widely used model system for endothelium dysfunction [43] associated with the pro-inflammatory response promoted by the secretome from ectopically senescing cells [44]. In fact, IR-induced PS is often associated with RT side effects such as tissue fibrosis, organ function disruption, and the elevation of secondary cancer risk as well as of cardiovascular disease incidence [45,46]. Figure 5 shows the occurrence of senescence, assessed by the histochemical staining of β-galactosidase (β-GAL) activity [47], both as an early (24 and 48 h) and as a delayed response (7 days) to the 0.5, 2, and 4 Gy of X-rays.

It can be seen that: (a) exposure to radiation alone (0.5 Gy−, 2 Gy− and 4 Gy− in Figure 5) causes cells to readily enter PS compared to the control unirradiated cells with no extracts, that is Ctrl −, which show physiological time-dependent senescence; (b) such a response occurs as early as 24 h after irradiation, persisting up to a week; (c) at each given time, the fraction of senescent cells increases with the X-ray dose. These data are in agreement with published results [48,49]. More interestingly, however, the OLC clearly appears to suppress the onset of PS at all times and for all doses investigated (Figure 5). In fact, among the OLC-treated irradiated HUVECs, the proportion of senescent cells also increases in time as a function of the radiation dose; however, it always stays significantly lower than that of their irradiated non-OLC-treated counterparts. Specifically, whereas, at 7 days post-irradiation, the level of PS reaches its maximum, ranging from 0.45 to about 0.55 in HUVECs exposed to 0.5 ÷ 4 Gy in the absence of the extract (Figure 5, green bars), the fraction of senescent cells among irradiated OLC-treated HUVECs never exceeds 0.4 at all times over the same dose interval. As mentioned above, treatment with the 12.5 μg/mL OLC alone (Ctrl +) did not exert any effect compared to the untreated unirradiated cells (Ctrl −), as shown by the almost identical levels of physiological senescence as time progressed.

#### 3.3.2. OLC Mitigates Radiation-Induced DNA Damage in Normal Cell Lines

**Figure 6:**
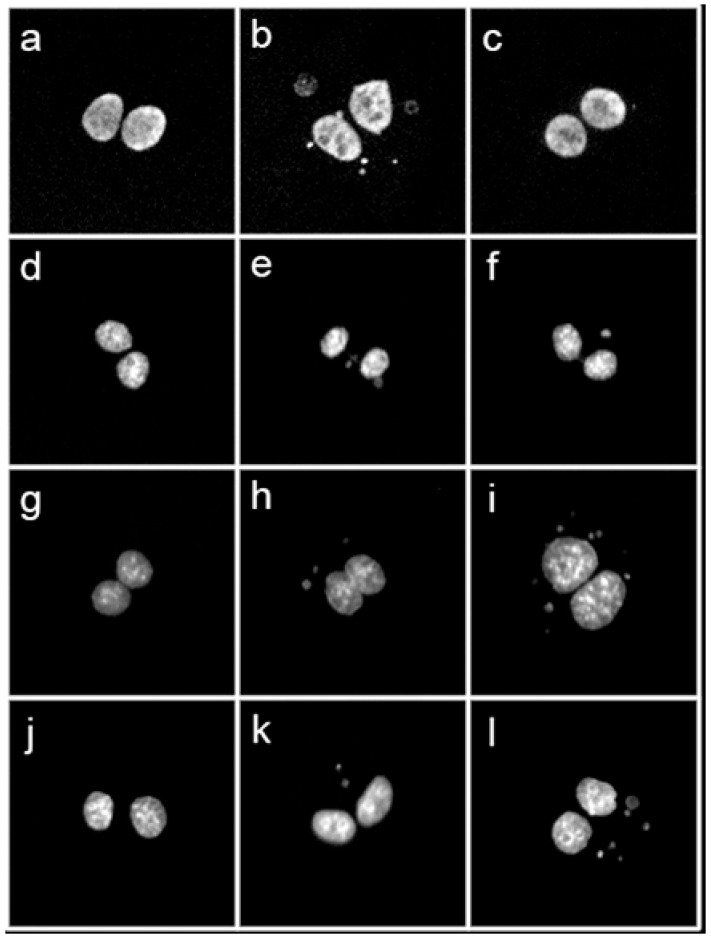
10× magnification micrograph showing the typology of observed DNA damage per cell line and treatment conditions (no OLC vs. OLC): (**a**,**d**) unirradiated binucleated (BN) cells from normal MCF-10A and HUVEC cell lines, respectively; (**b**,**e**) BN MCF-10A and HUVECs irradiated with 4 Gy of X-rays in the absence of extract presenting multiple micronuclei (MN); (**c**,**f**) BN MCF-10A and HUVEC irradiated with the same dose but after OLC treatment and showing just one MN in each BN cell; (**g**,**j**) BN cells from cancer DU-145 and PANC-1 cell lines, respectively; (**h**,**k**) BN cells from DU-145 and PANC-1 respectively, irradiated with 4 Gy of X-rays without extract and showing only a couple of MN; (**i**,**l**) BN cells from DU-145 and PANC-1, 4Gy-irradiated in the presence of OLC showing a considerable amount of DNA damage in the form of several MN per BN cell.

DNA damage elicited by X-ray irradiation was measured as the frequency of micronuclei (MN) in binucleated (BN) cells according to the well-established Cytokinesis-Block MicroNucleus (CBMN) assay [50]. MN represent portions of damaged DNA, either whole chromosomes or thereof acentric fragments, which fail to properly segregate in daughter nuclei and lag behind during cell division, hence the possibility to visualize them if cell division is arrested at the BN stage by means of cytochalasin B (CytB), an inhibitor of the actin polymerization required for the cytokinesis. Together with the analysis of radiation-induced structural chromosome aberrations (CA) that result from unrepaired double-strand breaks, the CBMN assay is one of the most reliable and widely used methods to quantify IR-associated genotoxicity given its relative simplicity and its sensitivity to reveal dose-dependent DNA breakage and/or chromosome loss following, as is the case for the CA [51,52], varying radiation exposure regimes [53,54]. The panel in Figure 6 shows typical images of BN cells, with or without MN, for the studied cell lines and the experimental conditions (exposure in the presence or the absence of the OLC), for the highest dose used, that is 4 Gy of X-rays.

**Figure 7:**
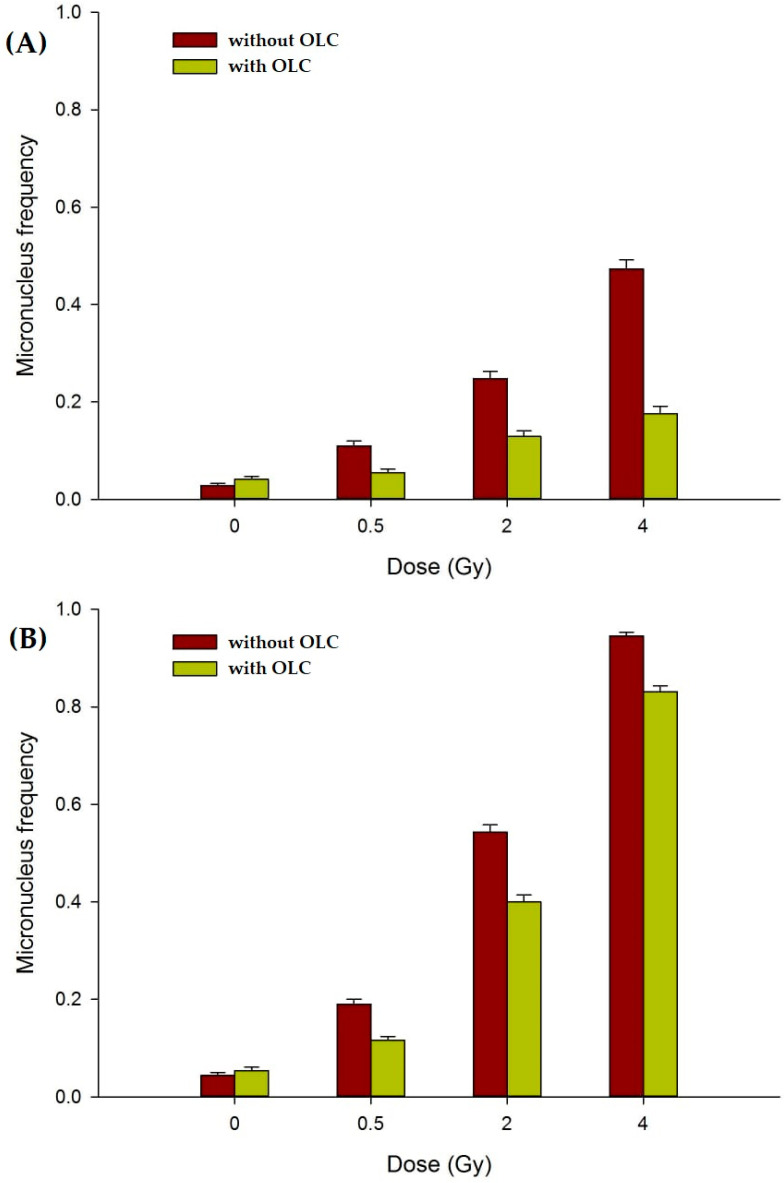
MN frequency in normal cell lines: (**A**) Dose-dependent MN induction in HUVECs; (**B**) Dose-dependent MN induction in MCF-10A cells. Irradiation in the presence of the OLC (green bars) yielded consistently less DNA damage compared to non OLC-treated samples (brown bars). MN frequency values are depicted as mean ± SE for at least two independent experiments per cell lines.

When the CBMN was performed on the two normal cell lines employed in this study, that is HUVECs and MCF-10A, the presence of the OLC resulted in a significant attenuation of the measured MN frequency, as shown in Figure 7. At the used concentration, the OLC did not cause damage per se in unirradiated samples: for example, at 0 Gy the MN frequency in HUVECs was 0.028 in non OLC-treated samples compared to a value of 0.041 measured for OLC-treated cells (*p* = 0.096). On the other hand, although the absolute frequency of DNA damage recorded in HUVECs (Figure 7A) was smaller compared to that observed in MCF-10 (Figure 7B), the presence of OLC markedly reduced the MN frequency in both cell lines, such a sparing effect proportionally greater at 2 and 4 Gy in HUVECs compared to MCF-10A. In fact, in HUVECs at 4Gy OLC suppressed X-ray-induced MN formation by more than two thirds (Figure 6a). At the same dose, the protection afforded by OLC in MCF-10A was less but still statistically different (Figure 7B), with a yield of 0.945 MN/cell in the absence of OLC compared to a value of 0.831 MN/cell in OLC-treated MCF-10A cells (*p* < 0.001). At the lowest dose used, that is 0.5 Gy, the OLC was able to almost exactly halve the damaged caused by radiation in both cells lines, reducing the MN frequency from 0.110 and 0.190 to 0.055 and 0.116 in HUVEC and MCF-10A, respectively. The overall lower MN frequency measured in HUVECs compared to epithelial MCF-10A cells could be due to their proneness to undergo PS as well as reflecting a sensitivity to apoptosis (the latter confirmed by frequent apoptotic bodies observed during microscopic scoring), both processes preventing a fraction of irradiated HUVECs to actively engage in the cell cycle and proceed through the first post-irradiation division, hence not reaching the BN stage. Exemplary images of undamaged MCF-10 and HUVEC BN cells (Figure 6a,d) can be compared to micronucleated BN cells for these cell lines, clearly showing more MN in MCF-10 cells and HUVECs following exposure to 4 Gy of X-rays in the absence of the extract (Figure 6b,e, respectively) compared to lesser damage expressed in BN cells following the same dose in the presence of the OLC (Figure 6c,f).

#### 3.3.3. The OLC Presence Exacerbates Radiation-Induced DNA Damage in Cancer Cells

**Figure 8:**
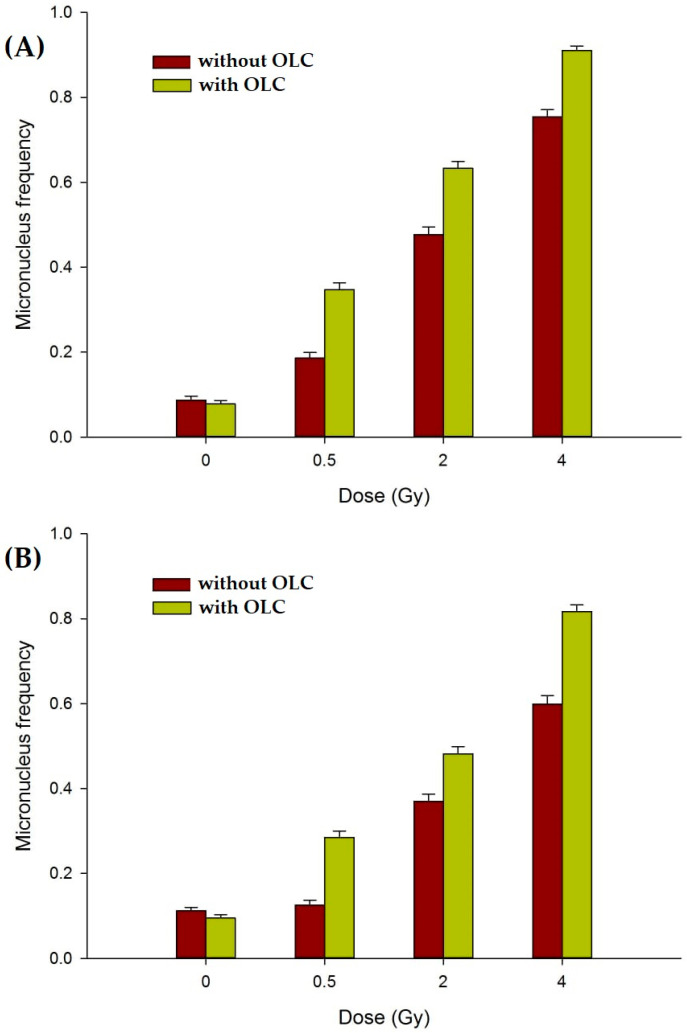
MN frequency in cancer cell lines: (**A**) Dose-dependent MN induction in prostate DU145 cancer cells; (**B**) dose-dependent MN induction in pancreatic PANC-1 cancer cells. Irradiation in the presence of the extract (green bars) causes more DNA damage compared to that measured in non OLC-treated samples (brown bars). Error bars represent SE from at least two independent experiments per cell line.

When prostate DU145 and pancreatic PANC-1 cancer cell lines were subjected to the same experimental conditions as those adopted for the non-cancer cell lines HUVEC and MCF-10A, that is 24-h treatment with, and irradiation in the presence of, 12.5 μg/mL OLC, the measured frequency of MN per BN cell was significantly higher than that observed in cancer cells irradiated without OLC (Figure 8). This clearly shows that OLC acts differently in terms of its ability to modulate X-ray-induced damage between cancer and normal cells. 

As also reported for normal cells (Section 3.3.2), the presence of OLC did not significantly add to the baseline MN level at 0 Gy. Such a baseline damage level was indeed slightly higher in DU145 and PANC-1 cells compared to normal HUVECs and MCF-10A cells, in keeping with the more pronounced genomic instability that characterizes cancer cells: an average of 0.10 MN per BN cell was found in unirradiated cancer cell samples, whether treated or untreated with OLC, compared to a baseline MN frequency of around 0.04 in non-cancer cells (Figure 7). Exposure to radiation led to an increase in such frequency in both cancer lines in a dose-dependent manner and to a greater extent in DU145 than in PANC-1 cells, in agreement with their greater radioresistance exhibited by pancreatic cancer [55]. Interestingly, in fact, not only does OLC enhance radiation damage in these cancer cells, but its radiosensitizing action is, on average, greater in the most radioresistant PANC-1 cells (Figure 7B): OLC leads to about a 1.8 to 1.2-fold increase in MN frequency in DU145 as X-ray doses increase from 0.5 to 4 Gy, whereas in PANC-1 cells such an enhancement factor ranges from 2.3 to 1.4 over the same dose interval. Thus, the presence of OLC in PANC-1 following 2 Gy of X-rays increases the mean frequency of MN per cell from 0.37 to almost 0.5. The capacity by OLC to exacerbate radiation-induced damage selectively in cancer cells can be appreciated by representative images of cancer cells exposed to 4 Gy (Figure 6), specifically showing an elevated mean number of MN in the extract-treated cells compared to those without in DU145 and PANC-1 cells (Figure 6i,l vs. Figure 6h,k, respectively).

## 4. Discussion

The chemical and metabolic profile of the crude leaf extract (OLC) of *O. europaea* L. cv. Caiazzana, an autochthonous cultivar from the Campania region (Italy) was investigated. The analysis confirmed the expected constitutive richness in oleuropein and its derivatives, mainly oleacein. The presence and content of the latter compound was related to olive tree genotypes, and, in olive drupes, levels were found to decrease during fruit maturation [56]. In leaves, it is reasonable to assume that there is a close connection to the harvest season and to the occurrence of oleuropein. Indeed, isotopic labelling of olive shoots showed its significant precursory role in the production of oleuropein and that this process was cultivar-, season-, and environment-dependent [57].

The putative radiomodulating properties of OLC were tested in vitro on a panel of cancer and normal cell lines. Our data seem to indicate that the same concentration of the investigated OLC exerts a differentially radioprotective action on normal cells while exhibiting radiosensitizing abilities in cancer cells over the X-ray dose range 0.5 ÷ 4 Gy. While previous studies had demonstrated the ability of phenolic secoiridoids, such as oleuropein, either to grant protection from IR deleterious effects due to its antioxidant properties or to enhance IR-induced damage owing to its pro-oxidant properties [23,24], this is the first report of an oleuropein derivatives-rich extract able to discriminate between cancer and normal cell in terms of their radioresponse under the same experimental conditions. Indeed, the ability to differentially act on healthy and cancer cells is the all-important criterion for clinically useful radioprotectors and radiosensitizers.

Even in the most innovative and technologically advanced RT approaches, increasing the therapeutic ratio continues to be a crucial factor [58,59]. The radiosensitivity of normal tissue and organs still remains the main dose-limiting factor in curative RT because of severe sequelae [2]. In addition, acquired/intrinsic cancer radioresistance is the underlying cause of recurrence and metastasization [7]. In this context, natural compounds have gained attention since they may abate IR adverse effects and enhance tumour radioresponse; in particular, food-isolable nutraceuticals show anti-proliferative and pro-oxidant effects on tumours [60,61] and antioxidant and anti-inflammatory activity in normal cells [62]. Such a dual behaviour advocates their use in RT since radiotoxicity mainly relies on DNA-damaging IR-induced reactive oxygen species (ROS). Despite a wealth of information on the bioactivity and cellular as well as in vivo effects of plant-derived polyphenols, specifically of *O. europaea*, as recently summarized by Olivares-Vincente et al. [63], only a few studies have addressed the effects oleuropein and oleuropein-derived compounds on radiation-induced cyto-genotoxicity as expressed by cellular senescence and MN formation, and even fewer where olive leaf extracts were tested.

The radiobiological endpoints chosen to investigate the OLC-mediated radiomodulation, that is premature senescence (PS) and DNA damage in the form of micronuclei (MN), are of clinical relevance. The accumulation of radiation-induced prematurely senescing non-cancer cells has been increasingly shown to impact normal tissue/organ homeostasis and function as well as promoting inflammatory responses. In fact, the endothelium integrity is of paramount importance as IR-induced inflammation may lead to RT-induced secondary cancers [4] and late cardiotoxicity [64]. At the same time, sublethally damaged normal cells pose a threat to genomic stability and contribute to elevate the risk of RT-induced secondary cancers. On the other hand, radiation-induced genotoxicity is the premise for cancer cell death and, ultimately, for successful RT outcome. Therefore, since in vitro MN induction is commonly considered as a reliable measure of IR effectiveness at damaging the DNA, its mitigation in normal cells is an indication of radioprotection whereas its elevation in cancer cells reflects radiosensitization.

The presence of OLC very effectively lowered the fraction of HUVECs undergoing PS as a result of X-ray exposure (Figure 5), and such a protecting effect was consistently shown at all doses and at each time point when cells were assayed. Recently, polyphenols have been considered to be of potential therapeutic importance for their ability to actually promote cancer cell senescence via several molecular targets, including oncogene regulation, the activation of DNA Damage Response (DDR), and other stress-related pathways [65]. However, equally compelling evidence exists in support of the anti-senescence action by polyphenols and their derivatives on several skin-derived cultured cells such as keratinocytes, melanocytes, and fibroblasts [66]; moreover, Menicacci et al. [67] showed that oleuropein aglycone from extra-virgin olive oil caused a significant reduction in β-gal positive cells and in the expression of the senescence-associated p16 in pre-senescent human lung (MRC-5) and neonatal dermal fibroblasts. Despite seemingly participating in the disruption of several pathways leading to PS, the main molecular anti-senescence mechanisms by polyphenols appear to hinge on their ability to counteract ROS production and pro-inflammatory responses [68]. A very recent work by Frediani et al. [69] showed that the deglycosylated product of oleuropein and hydroxytyrosol from olive fruit decreased the level of senescence elicited by very high doses of IR (6–8 Gy) suppressing the Senescence-Associated Secretory Phenotype. Our results therefore seem to confirm such findings proving that the OLC tested in this study can already exert an anti-senescence protective effect at doses as low as 0.5 Gy.

Our data showing an OLC-mediated reduction in radiation-induced genotoxicity, expressed in the form of MN scored in normal BN cells (Figure 6), are in keeping with similar findings that have been scantly reported over a long time on the radioprotection afforded by polyphenols to irradiated cellular DNA using the same endpoint. Thus, while, almost twenty years ago, Greenrod and Fenech [70] had assessed the impact of several wine-derived polyphenols on human lymphocytes irradiated in vitro by the CBMN assay reporting an anti-genotoxic action, Alcaraz et al. [71] very recently reported on the radioprotective effect by flavonoids in mice whole-body irradiated with 0.5 Gy of γ-radiation assessed by MN formation in polychromatic erythrocytes. More specifically, another recent work by Amani et al. [72] found that 100 mM of oleuropein afforded protection from apoptosis, clastogenicity, and genotoxicity, with the latter measured by MN occurrence, in human cultured lymphocytes exposed to a single dose of 2 Gy of γ-rays.

The effect of the same concentration of OLC granting DNA radioprotection in normal cells was instead reversed when cancer cells were treated with, and irradiated in the presence of, the extract: a significant radiosensitization manifested itself as an increase in the frequency of MN per BN cell following X-ray irradiation in two cancer cell lines, as shown in Figure 7. In a very recent review, Zhang et al. [73] analysed the most up-to-date data on the anti-cancer properties of oleuropein; however, apart from the already cited work by Xu and Xiao [24] on the radiosensitization of nasopharyngeal carcinoma, only one study on the effects of oleuropein can be found showing that it led to the suppression of ovarian cancer cell hypoxia-mediated radioresistance in a xenograft model [74]. Indeed, oleuropein alone, that is, without concomitant exposure to radiation, was tested on one of the two cancer cell lines used in this study, that is in prostate DU145 cancer cells, where it was shown to inhibit proliferation via an increase in ROS production [75].

There exists consensus around the notion that, at the heart of the double-edged geno-protective or geno-toxic potential of polyphenols, and therefore of oleuropein-based compounds, there ought to be an anti- or pro-oxidant activity that is very often concentration-dependent. As concentration increases, such extracts tend to switch from effective ROS scavengers to potent pro-oxidant agents. This view is reinforced by the few studies carried out in the presence of IR, which is a powerful source of ROS. However, to the best of our knowledge, this is the first report showing the ability by the same concentration of an extract from olive leaves to exert the radiomodulating ability differentially between normal and cancer cells. A similar result was recently published by Bektay et al. [76], who found that 0.25 mg/mL of olive leaf extracts selectively inhibited the cell viability of a rat liver cell line, sparing a healthy clone of rat liver cells. However, no radiation was used in this study. Our results may therefore have important implication in RT scenarios to widen the therapeutic window using a toxicity-free nutraceutical approach but warrant further in vitro and in vivo studies to elucidate the exact molecular mechanisms underlying the dual behaviour exhibited by the same concentration of the OLC tested in our study. One possibility for such selectivity may be related to the intracellular ROS (iROS) content, which tends to be physiologically different between normal and cancer cells, with the latter being able to generate more iROS and with the known ability of oleuropein to suppress the NF-kB signalling pathway involved in a variety of important cellular processes in many cancer cells. Inhibition of NF-kB activation results in an increase in TNFα-induced ROS production, lipid peroxidation, and protein oxidation. Radiation would add to such a higher “background” oxidative stress in cancer cells. As suggested by Alcaraz et al. [71], under certain conditions depending, among other factors, on the number and positions of hydroxyl groups, polyphenolic compounds through the chelation of chromosome-associated metal ion Cu^2+^ can switch from an anti-oxidant to a pro-oxidant activity, a mechanism already postulated to explain flavonoid-mediated DNA damage [77]. Thus, it is possible that, in normal cells, the additional level of ROS produced by radiation can be dealt with effectively by the anti-oxidant scavenging ability of the oleuropein-rich OLC at the concentration used in this study, whereas the peculiar redox status of cancer cells combined with the mentioned properties of oleuropein and the additional radiation-induced ROS could reverse the same amount of OLC to an effective pro-oxidant agent. Interestingly, none of the compounds tentatively identified in the OLC tested in this study appear to be associated with the modification of known target gene expression in pancreatic or prostate cancer cells, as reported in an extensive biodatabase (NaturaProDB) recently compiled by Theofylaktou et al. [78]. This therefore adds to the novelty of our findings and warrants further studies on the radiomodulating properties of the *Olea europaea* L. cv. Caiazzana leaf extract at both the genetic and epigenetic levels to elucidate its precise mode of action. Furthermore, preclinical and clinical studies will be needed to verify the efficacy of the OLC single radiomodulating dose. In this context, it has been shown that olive leaf phenolic extracts or their formulas increase the bioavailability of hydroxytyrosol and oleuropein in both mice and humans [79,80]. In fact, although conducted on a small number of volunteers, quantification studies of the bioavailability and metabolism of the two most attentive compounds of the olive leaf have shown that the formulation of the extract and the delivery method influence absorption and metabolism [80]. Indeed, any future study must strictly consider the chemical composition of OLC, which is particularly rich in oleacenin and its derivatives. Unfortunately, knowledge in this regard is extremely limited to date, with the exception of one study aimed at quantifying the distribution of oleacenin, administered in rats in a single dose (2.5 mL/300 g body weight) by gavage, in plasma and several organs, including stomach, small intestine, liver, heart, spleen, thyroid, lung, brain, kidney, and skin [81].

## 5. Conclusions

We have, for the first time, chemically and radiobiologically profiled the olive leaf Caiazzana (OLC) crude extract obtained from a cultivar (Caiazzana) autochthons to Regione Campania, in Southern Italy. The metabolic analysis of its polyphenolic content showed that it is rich in oleacein and its esters, which, reasonably, through their hydroxytyrosol moiety, allowed OLC to exert an important antiradical and to reduce efficacy. Interestingly, the extract exhibited peculiar in vitro radiomodulating properties, being capable, for one identical concentration, to exert a differential action, exacerbating the DNA damage inflicted by graded doses of X-rays in the two tested cancer lines, whilst mitigating the cyto- and genotoxicity caused by such doses in the two normal cell lines. To our knowledge, this behavior is unique. In fact, it is well known that plant polyphenols may either act as radiosensitizers or radioprotectors; however, no study has thus far reported such a dual, cell-type-specific action using the same compound concentration. Such selectivity between tumour and non-cancer cells bears important implications, if further confirmed, for its potential usefulness to widen the radiotherapy therapeutic window. Finally, the sustainability of the extraction process from pruned leaves, normally regarded as waste, adds value to the possible societal impact of our findings.

## Figures and Tables

**Table 1:** TOF-MS and MS/MS data of compounds tentatively identified in OLC extract. (RDB = Ring and Double Bond. Base peaks are reported in bold).

Peak n.	RT (min)	Tentative Assignment	Formula	[M-H]^−^calc. (*m*/*z*)	[M-H]^−^ Found (*m*/*z*)	Error (ppm)	RDB	HR-MS/MS Fragment Ions (*m*/*z*) (Base Peaks in Bold)
**1**	0.316	Hexitol	C_6_H_14_O_6_	181.0718	181.0719	0.8	0	181.0722; 163.0636; 136.8708; 119.0352; 113.0240; **101.0241**; 89.0245
**2**	0.326	Quinic acid	C_7_H_12_O_6_	191.0561	191.0565	2.0	2	**191.0558**; 173.0447; 127.0403; 109.0293; 93.0348
**3**	1.651	Hydroxytyrosol	C_8_H_10_O_3_	153.0557	153.0557	0.0	4	153.0561; **123.0454**
**4**	1.835	Hydroxytyrosol hexoside	C_14_H_20_O_8_	315.1085	315.1084	−0.4	5	315.1081; 153.0556; **135.0454**; 123.0453; 119.0352; 119.0352; 113.0251; 101.0252; 89.0254
**5**	3.375	12-hydroxyjasmonate sulfate	C_12_H_18_O_7_S	305.0700	305.0702	0.5	4	305.0705; 225.1135; 174.9560; 147.0816; 130.9665; **96.9606**
**6**	4.509	Unknown	C_16_H_26_O_10_	377.1453	377.1454	0.2	4	377.1455; 197.0824; **153.0925**
**7**	5.329	Phenethyl primeveroside	C_19_H_28_O_10_	415.1610	415.1609	−0.2	6	191.0557; 179.0566; 161.0455; 149.0458; 131.0359; 119.0356; 113.0245; 101.0252; **89.0250**
**8**	6.377	2-(2-ethyl-3-hydroxy-6-propionylcyclohexyl) acetic acid glucoside	C_19_H_32_O_9_	403.1974	403.1977	0.9	4	403.1992; 241.1448; **223.1346**; 161.0463; 119.0355; 113.0252; 101.0253; 89.0247
**9**	6.482	Luteolin di-hexoside	C_27_H_30_O_6_	609.1461	609.1463	0.3	13	609.1486; **447.0949**; 285.0403
**10**	7.789	Rutin	C_27_H_30_O_6_	609.1461	609.1472	1.8	13	**609.1482**; 301.0347; 300.0271; 271.0229
**11**	8.058	Luteolin hexoside 1	C_21_H_20_O_11_	447.0933	447.0935	0.5	12	447.0945; **285.0405**;284.0329
**12**	8.063	Verbascoside	C_29_H_36_O_15_	623.1981	623.1983	0.2	12	**623.1999**; 461.1665; 161.0243
**13**	8.958	Oleacein	C_17_H_20_O_6_	319.1187	319.1190 639.2455 [2M-H]^−^	0.9	8	183.0670; 165.0561; 139.0772; 123.0454; 113.0251; **95.0512**
**14**	9.194	Quercetin deoxyhexoside	C_21_H_20_O_11_	447.0933	447.0931	−0.4	12	447.0950; 301.0359; **300.0279**; 271.0246; 255.0290; 178.9986; 151.0036
**15**	9.437	Lipedoside A isomer 1	C_29_H_36_O_14_	607.2032	607.2029	−0.5	12	**607.2027**; 461.1684; 163.0400; 145.0296
**16**	9.450	Apigenin hexosyldeoxyhexoside 1	C_27_H_30_O_14_	577.1563	577.1563	0	13	**577.1575**; 269.0447; 268.0371
**17**	9.551	Luteolin hexoside 2	C_21_H_20_O_11_	447.0933	447.0940	1.6	12	**285.0403**
**18**	9.588	Oleuropein hexoside	C_31_H_42_O_18_	701.2298	701.2287	−1.6	11	701.2339; **539.1800**; 469.1362; 437.1110; 377.1253; 307.0811; 275.0923; 179.0567
**19**	9.620	Lucidumoside B	C_25_H_34_O_13_	541.1927	541.1931	0.9	9	361.1293; **225.0770**; 193.0504; 181.0872; 149.0606; 121.0659; 89.0248
**20**	9.873	Apigenin hexosyldeoxyhexoside 2	C_27_H_30_O_14_	577.1563	577.1577	2.5	13	577.1577; **269.0450**
**21**	9.865	Lipedoside A isomer 2	C_29_H_36_O_14_	607.2032	607.2044	1.9	12	**607.2049**; 461.1679; 163.0404; 145.0295
**22**	10.183	Oleuropein	C_25_H_32_O_13_	539.1770	539.1784	2.6	10	539.1786; 403.1240; 377.1237; 345.0975; 327.0874; 307.0823; **275.0911**; 223.0605; 179.0564; 149.0247; 95.0509
**23**	10.343	Luteolin hexoside 3	C_21_H_20_O_11_	447.0933	447.0930	−0.6	12	447.0929; **285.0406**
**24**	10.539	Diosmin	C_28_H_32_O_15_	607.1668	607.1679	1.7	13	**607.1689**; 299.0552; 284.0311
**25**	12.136	Luteolin	C_15_H_10_O_6_	285.0405	285.0406	0.5	11	**285.0413**; 217.0507; 199.0402; 175.0403; 151.0038; 133.0300; 107.0140
**26**	13.213	Oleacein dimethyl acetal	C_19_H_26_O_7_	365.1606	365.1603	−0.8	7	**229.1086**; 211.0976; 201.1133; 185.1185; 169.0868; 153.0922; 138.0687; 121.0665
**27**	14.273	Apigenin	C_15_H_10_O_5_	269.0455	269.0453	−0.9	11	**269.0453**; 225.0550; 201.0554; 183.0445; 181.0664; 159.0453; 151.0037; 149.0241; 117.0347; 107.0141
**28**	14.999	Diosmetin	C_16_H_12_O_6_	299.0561	299.0561	0.0	11	299.0559; **284.0321**; 256.0374
**29**	15.313	Oleacein ethylmethyl acetal	C_20_H_28_O_7_	379.1762	379.1769	1.8	7	**243.1243**; 225.1138; 215.1285; 199.1338; 167.1075; 153.0921; 138.0690; 121.0659
**30**	17.198	Oleacein diethyl acetal	C_21_H_30_O_7_	393.1919	393.1925	1.6	7	**257.1399**; 239.1289; 229.1446; 213.1497; 167.1080; 139.0766; 121.0664
**31**	17.671	Oleuropein derivative	C_36_H_44_O_18_	763.2455	763.2461	0.8	15	693.2048; **539.1785**; 461.0730; 377.1250; 307.0829; 275.0904; 149.0237
**32**	25.740	Oleanolic acid	C_30_H_48_O_3_	455.3531	455.3527	−0.9	7	**455.3531**; 407.3318; 405.3162; 373.2506; 345.2192

## Data Availability

The data is contained within the article and Supplementary Materials.

## Supplementary Materials

The following supporting information can be downloaded at: https://www.mdpi.com/article/10.3390/antiox11081603/s1, Figure S1: (a) Total Ion Current (TIC) and (b) Base Peak Chromatogram (BPC) of OLC extract. Red arrows denote the quantified compounds, considered the main responsible for the observed bioactivity; Figure S2: TOF-MS/MS spectra of (a) hydroxytyrosol and (b) hydroxytyrosol hexoside; (c) hypothesized fragmentation pathways; Figure S3: TOF-MS/MS spectra of compounds 12 (at *m*/*z* 623.1981, calc. mass), 15 and 21 (at *m*/*z* 607.2032, calc. mass). The hypothesized chemical structures of the detected ions are reported with theoretical *m*/*z* values; Figure S4: TOF-MS/MS spectra of detected flavonoid glycosides. The aglycone structures are provided together with the glyconic moieties. Figure S5: TOF-MS spectra of flavones: (a) luteolin, (b) apigenin, and (c) diosmetin; Figure S6: Experimental evidence of the occurrence of oleanolic acid (XIC = eXtracted Ion Chromatogram).
