# Supplementary material for: Differential Radiomodulating Action of Olea europaea L. cv. Caiazzana Leaf Extract on Human Normal and Cancer Cells: A Joint Chemical and Radiobiological Approach
Source: Antioxidants (Basel). 2022 Aug 19;11(8):1603. doi: 10.3390/antiox11081603 (PMC9404970; doi:10.3390/antiox11081603)
Supplement: Supplementary file 1 [file antioxidants-11-01603-s001.zip › antioxidants-1827432-SI.pdf]

Supplementary materials

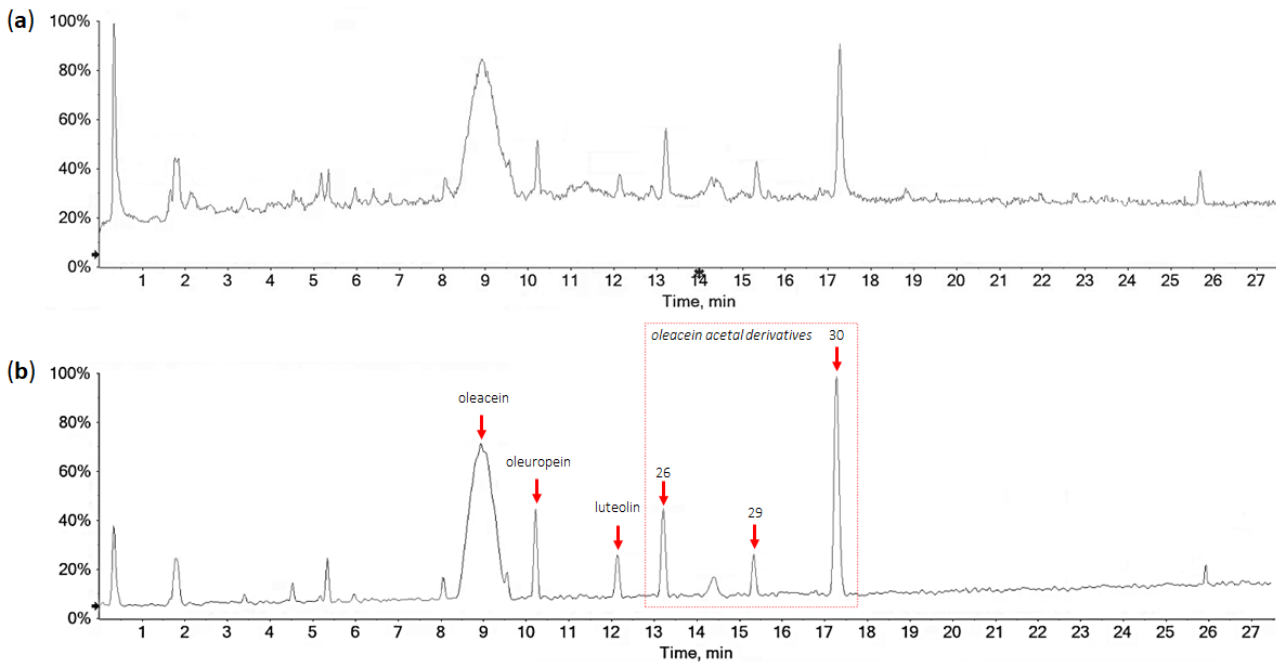

Figure S1. (**a**) Total Ion Current (TIC) and (**b**) Base Peak Chromatogram (BPC) of OLC extract. Red arrows denote the quantified compounds, considered the main responsible for the observed bioactivity.

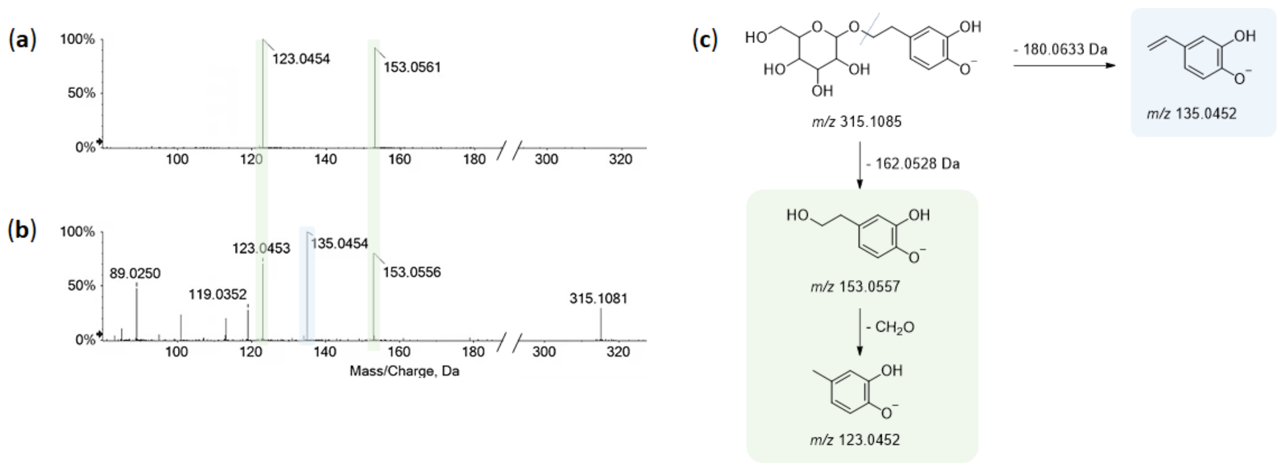

Figure S2. TOF-MS/MS spectra of **(a)** hydroxytyrosol and **(b)** hydroxytyrosol hexoside; **(c)** hypothesized fragmentation pathways.

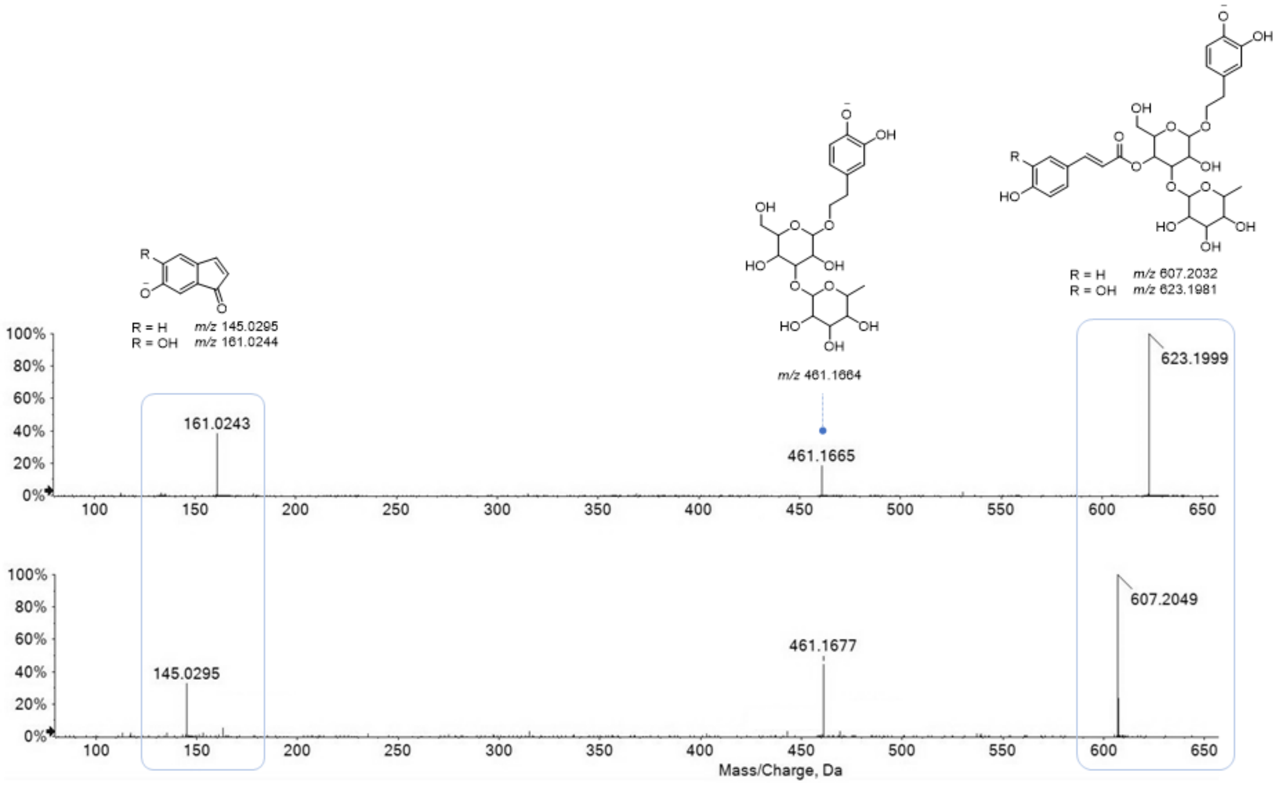

Figure S3. TOF-MS/MS spectra of compounds **12** (at *m/z* 623.1981, calc. mass), **15** and **21** (at *m/z* 607.2032, calc. mass). The hypothesized chemical structures of the detected ions are reported with theoretical *m/z* values.

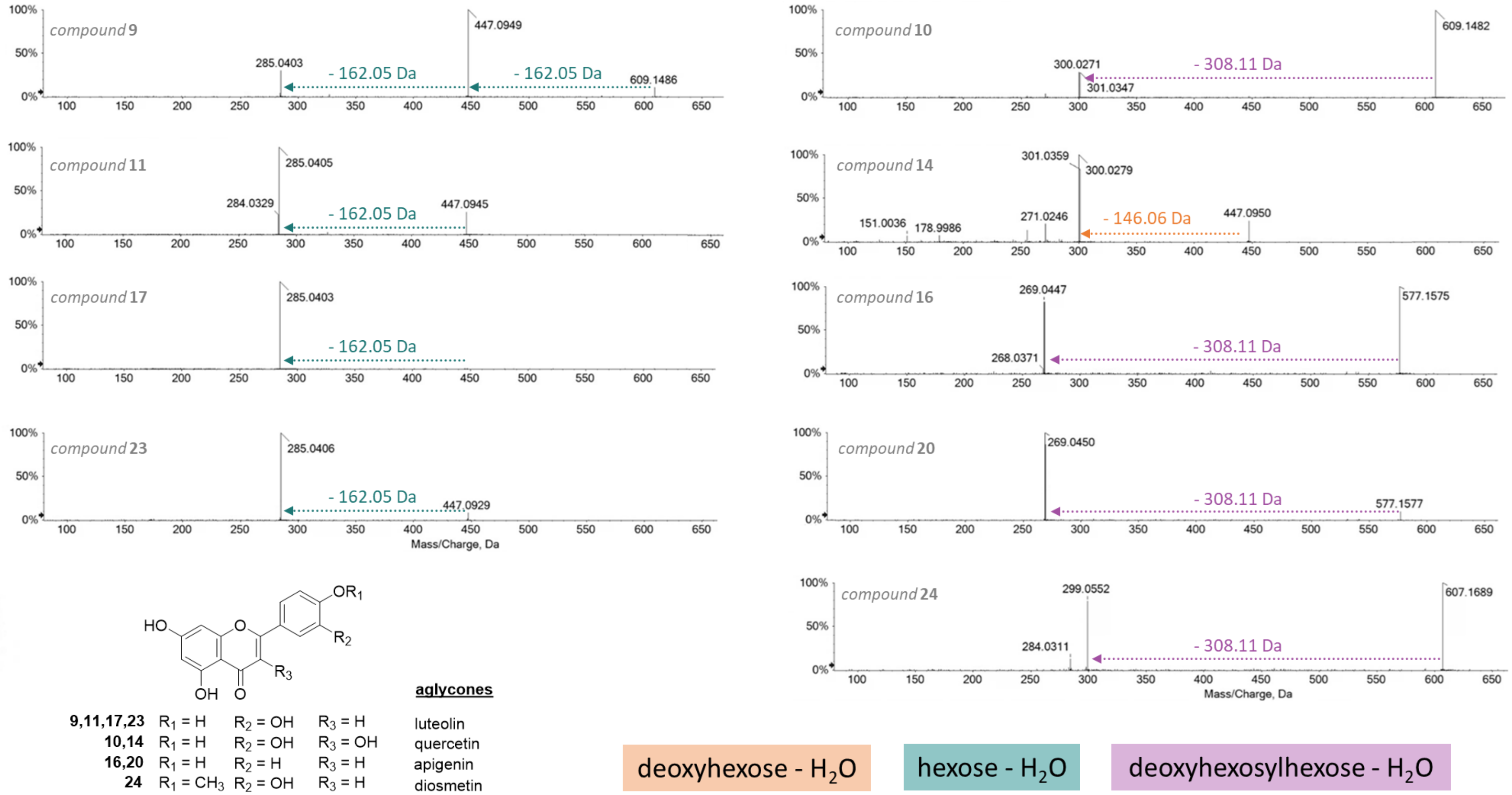

Figure S4. TOF-MS/MS spectra of detected flavonoid glycosides. The aglycone structures are provided together with the glyconic moieties.

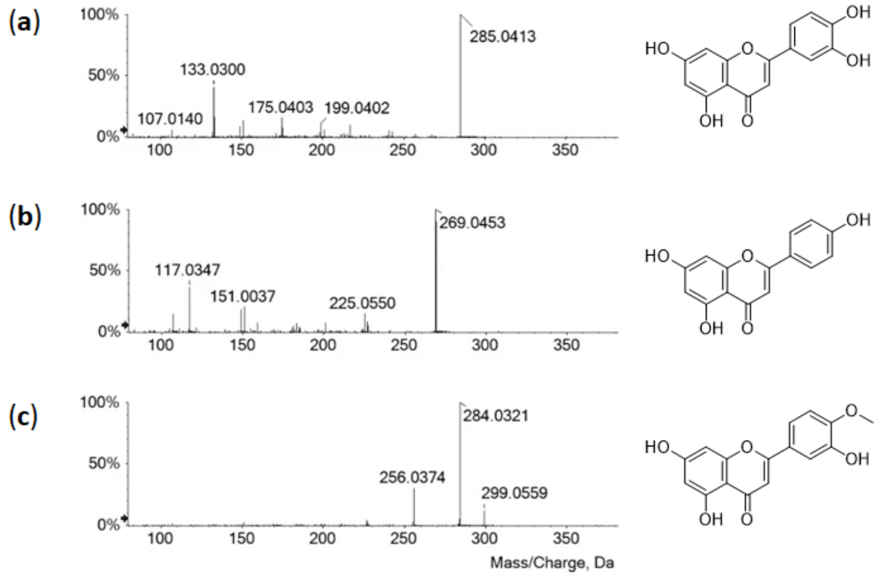

Figure S5. TOF-MS spectra of flavones: (**a**) luteolin, (**b**) apigenin, and (**c**) diosmetin.

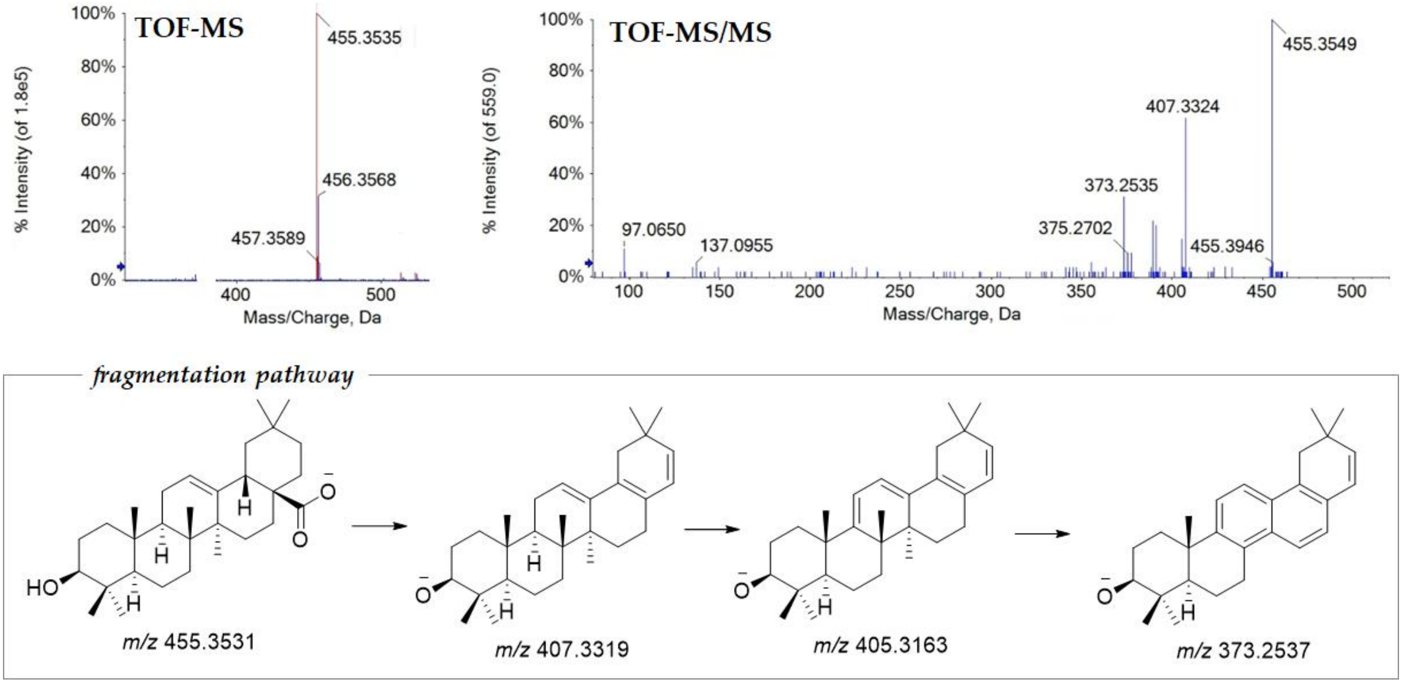

Figure S6. Experimental evidence of the occurrence of oleanolic acid (XIC = eXtracted Ion Chromatogram).
